# *Pasung*: Physical restraint and confinement of the mentally ill in the community

**DOI:** 10.1186/1752-4458-2-8

**Published:** 2008-06-16

**Authors:** Harry Minas, Hervita Diatri

**Affiliations:** 1 Centre for International mental Health, Melbourne School of Population Health, The University of Melbourne, Parkville, Victoria 3010, Australia; 2 Department of Psychiatry, University of Indonesia, Kimia II No. 35, Center Jakarta, 10430, Indonesia

## Abstract

**Background:**

Physical restraint and confinement (*pasung*) by families of people with mental illness is known to occur in many parts of the world but has attracted limited investigation. This preliminary observational study was carried out on Samosir Island in Sumatra, Indonesia, to investigate the nature of such restraint and confinement, the clinical characteristics of people restrained, and the reasons given by families and communities for applying such restraint.

**Methods:**

The research method was cross-sectional observational research in a natural setting, carried out during a six-month period of working as the only psychiatrist in a remote district.

**Results:**

Fifteen cases of *pasung*, approximately even numbers of males and females and almost all with a diagnosis of schizophrenia were identified. Duration of restraint ranged from two to 21 years.

**Discussion and Conclusion:**

The provision of basic community mental health services, where there were none before, enabled the majority of the people who had been restrained to receive psychiatric treatment and to be released from *pasung*.

## Background

"All persons with a mental illness... shall be treated with humanity and respect for the inherent dignity of the human person."

UN Resolution 46/119[1]

In Indonesia the term *pasung *refers to the physical restraint or confinement of "criminals, crazy and dangerously aggressive people." [2]

For six months during 2006 HD worked as the only psychiatrist on Samosir Island in North Sumatra. Nearly the size of Singapore, Samosir has a population of approximately 130,000. It is the world's largest island within an island, located in Lake Toba, in the planet's largest caldera (collapsed volcano formation). Although Samosir is a popular tourist destination most of the population, predominantly ethnic Batak, is rural and poor.

Samosir has a reasonably well-developed district health service with a district general hospital and 11 community health centres (*puskesmas*), each with two or three doctors.

However, like most rural districts in Indonesia, Samosir has no mental health budget or mental health service. The nearest available mental health service is the mental hospital in Medan, approximately six hours travel by boat and then by road.

Soon after arriving on Samosir HD became aware of cases of *pasung *in this small but widely dispersed community. The objective of this work was to locate all cases of *pasung *on Samosir Island, to investigate the circumstances of restraint, and to release from restraint as many as was possible.

## Methods

The research method was cross-sectional observational research in a natural setting, carried out during a six-month period of working as the only psychiatrist in Samosir, with some limited follow-up.

Case finding was through discussion with doctors and other clinical staff in the 11 *puskesmas*, visiting villages and having discussions with village heads and other key members of village communities.

Diagnosis, and the other information reported in this paper, was based on interviews with the person who was restrained, with members of the family when they were available and other members of the village communities who could give relevant information. In those cases where release from *pasung *was possible and treatment was initiated limited follow-up information is available from repeat visits to the home village.

## Results

**Figure 1:**
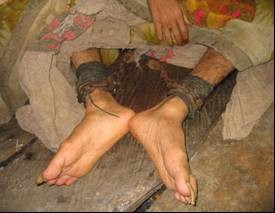
Iron shackles are fixed to the wooden floor of a hut in which the person is confined.

**Figure 2:**
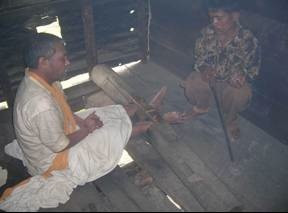
This man has his ankles in wooden stocks.

Over the course of six months 15 cases of *pasung *were identified. Figures 1 and 2 show typical forms of restraint, with iron shackles and wooden stocks. A summary of the characteristics of the cases found is presented in Table 1.

**Table 1:** Cases of *Pasung *on Samosir Island

**Case Number**	**Male/Female**	**Age (Years)**	**Diagnosis**	**Duration of illness (years)**	**Previous psychiatric treatment (Yes/No)**	**Type of *pasung***	**Duration in *pasung *(years)**	**Initiator**	**Reason for pasung**	**Released? (Yes/No)**
1	M	56	Depression, post-schizophrenia	>25	yes	wooden stocks	21	Family and community	History of violence; treatment not affordable	yes
2	M	26	Schizophrenia	5	yes	confined in a small room	5	Family	Treatment not affordable; fear of violence	yes
3	M	28	Schizophrenia and suspected mild mental retardation	10	yes	tied with rope	10	Family	Treatment not affordable; fear of violence; fear that he will get lost and come to harm	yes
4	M	27	Schizophrenia	12	no	chained	10	Family and community	Setting fire to family house and church; history of violence; considered dangerous	no
5	F	45	Schizophrenia	>15	no	confined in a small room	4	family	Fear that she will run away and come to harm	yes
6	F	55	Schizophrenia	10	yes	confined in a small room	2	family-her children	Treatment not affordable; fear that she will run away; fear of possibility of suicide	yes
7	F	41	Schizophrenia	16	no	tied with rope	16	family	Treatment not affordable; fear that she will run away and come to harm; inappropriate sexual behaviour; no-one to look after her	yes
8	F	32	Schizophrenia	5	yes	confined in a small room	3	family	Treatment not affordable; fear that she will run away and come to harm; no-one to look after her	yes
9	M	27	Schizophrenia	3	yes	confined in a small room	2	family	History of violence; treatment not affordable; no-one to look after him	yes
10	M	25	Schizophrenia	5	no	confined in a small room	5	family	History of violence	yes
11	F	25	Schizoaffective disorder	5	no	confined in a small stall in the rice-field	5	family	Treatment not affordable; fear that she will run away and come to harm; wasting money; inappropriate sexual behaviour	yes
12	F	56	Temporal lobe epilepsy with interictal hallucinations and personality change	>20	yes	tied with rope	15	family	History of violence; treatment not affordable; fear that she will come to harm; no-one to look after her	yes, temporary
13	F	55	Dementia of unknown aetiology with behavioural disturbance	5	yes	tied with rope	5	family	Treatment not affordable; fear that she will come to harm and come to harm through accident; no-one to look after her	yes, temporary
14	M	26	Schizophrenia and suspected mild mental retardation	5	no	wooden stocks	3	family	History of violence; considered to be dangerous to others; no-one to look after him; treatment not affordable	yes
15	M	35	Schizophrenia	15	yes	wooden stocks	6	Family and community	History of violence; considered to be dangerous to others, no-one is willing to look after him; refuses treatment	no

Of the 15 cases eight were male and seven female, ranging in age from 25 to 56 years. In 13 of the cases a diagnosis of schizophrenia was made. In one case (case 12) the diagnosis was personality change and interictal hallucinations due to probable temporal lobe epilepsy, and in another (case 13) the diagnosis was dementia of unknown aetiology with behavioural disturbance. Duration of illness ranged from three to more than 25 years. Somewhat surprisingly nine of the 15 cases had had previous psychiatric treatment. The commonest reason given for the discontinuation of treatment was that it was unaffordable. The major component of the cost of treatment that could not be afforded was the cost of travel, since the nearest place at which psychiatric treatment was available was in the city of Medan, six hours away by boat and then road.

The most common form of *pasung *was confinement in a small room or hut. In one case the hut was in the middle of the family's rice field some ten minutes walk from the house. In all cases the family (sometimes with the support of other members of the community) had initiated and was responsible for maintaining the *pasung*. Duration of *pasung *ranged from two to 21 years.

The reasons given for *pasung *were often multiple, including violence, concern about the person wandering off or running away and coming to harm, concern about possibility of suicide, and the unavailability of a caregiver. In seven cases – six male and one female – the primary reason was a history of, or concern about possible, violence. A 26 year old man had attacked his father with a knife, and a 35 year old man had attempted to strangle a priest. A 27 year old man had murdered three people and had set fire to the family house and to the village church. During the six months that he was in prison the prison authorities realised he was mentally ill at which point he was returned to his family, with no formal psychiatric assessment or treatment.

**Figure 3:**
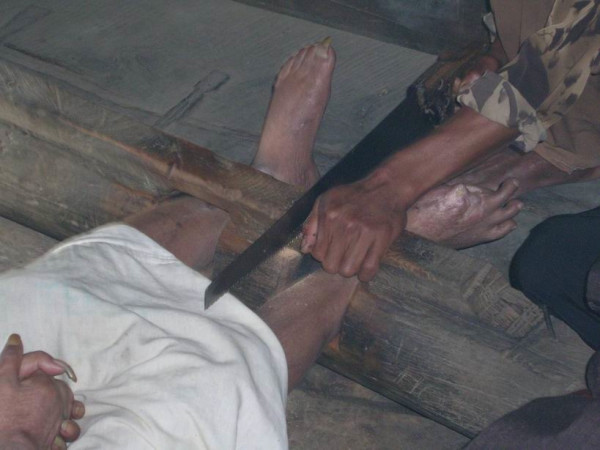
The man shown in Figure 2 being released from *pasung*.

Following assessment of risk, provision of information to the family and to the village head and the village community, initiation of treatment, and provision of basic education to the *puskesmas *doctors and nurses, it was determined that thirteen of the 15 cases could be treated and released from *pasung *(Figure 3). In six of the 13 cases that were treated treatment was initiated during a short hospital admission and then continued after discharge. In seven cases outpatient treatment was deemed to be appropriate. At follow-up, at the end of the six month period, only two of the 13 cases had been again restrained.

## Discussion

Physical restraint of people with mental illness has a long and inglorious history. Philippe Pinel is credited with having released the mentally ill from their chains at the Bicêtre and the Salpêtrière hospitals in Paris at the end of the 18^th ^century. And yet physical restraint has continued in mental hospitals,[3] in religious shrines and healing sanctuaries, [4-8] and other settings in many parts of the world. [8-14] Such restraint, including shackles, rope, stocks,[15] cages,[11] and being locked in confined spaces, is applied to men, [16-18] women[19] and children[20]. The practice seems to have aroused little human rights concern, except when mentally ill people in chains have died.[4,17,21]

Several findings from this study should be highlighted. First, it is families that are responsible for restraint and confinement. The main motivations for the practice are to prevent harm to others and to the ill person. Second, more than half of the ill people had had previous psychiatric treatment that had been discontinued, almost always because of the unavailability of affordable treatment. When affordable treatment was offered almost all patients and families accepted the treatment. Third, despite the fact that many of the people had been in *pasung *for many years, all except two of the 13 who were released were still free at follow-up. The final and most important point is that achieving a favourable outcome for the majority of people in *pasung *required the development of a basic mental health system for Samosir.

The development of a basic mental health service had the support of the District Health Office and the District Parliament. An inpatient facility (one room with two beds) and an outpatient unit were established in the District General Hospital. The availability of psychotropic drugs was improved and training was carried out for the *puskesmas *doctors, nurses and midwives. Information was provided to village heads and the families of people with serious mental illness. A small mobile team, consisting of a general nurse, a midwife and a social worker from the District Health Office, supported by *puskesmas *doctors, nurses or midwives, was able to visit mentally ill people in their homes.

The phenomenon of restraint in the community of people with mental illness has been surprisingly neglected by researchers. There is a need for two types of research. The first is epidemiological research that will estimate the prevalence of the practice in different countries and different settings within countries, and that will clarify the role of a number of potential determinants, including poverty, potentially relevant social and cultural variables, and the availability and cost of psychiatric treatment and care. The second is ethnographic research that will elucidate the social and cultural meanings of the practice in a variety of settings and cultures, the relevance of beliefs about mental illness and psychiatric treatment, and the deep connections between poverty and *pasung*.

More surprisingly, the phenomenon has been largely neglected by human rights organizations and activists, and by development agencies and practitioners. The research that is referred to in the preceding paragraph will inform the development of international and local actions that have as their goal the eradication of this deeply offensive practice.

## Conclusion

The findings that are reported in this study demonstrate that the abuse of human rights that *pasung *represents is not a product of the callousness or ignorance of families and communities, or by refusal to accept psychiatric treatment, but may more correctly be attributed to neglect by governments of their responsibility to provide basic mental health services for people with severe mental illness. Systematic strategies need to be developed to eradicate this practice. This will require the collaborative participation of policy makers, service developers and managers and health professionals, development NGOs and bilateral agencies, and civil society organisations including those with a clear focus on promotion and protection of human rights of the most vulnerable in low resource settings. In the end, the only effective and sustainable strategy for eradicating the practice is to ensure that families and communities have affordable and equitable access to basic mental health services.

## Competing interests

The authors declare that they have no competing interests.

## Authors' contributions

HD collected the data, HM conceived and took primary responsibility for the writing of the manuscript, HM and HD jointly interpreted the data. Both authors have approved the final version of the manuscript.
